# Supplementary material for: Study of Physicochemical Quality and Organic Contamination in Algerian Honey
Source: Foods. 2024 May 4;13(9):1413. doi: 10.3390/foods13091413 (PMC11083514; doi:10.3390/foods13091413)
Supplement: Supplementary file 1 [file foods-13-01413-s001.zip › foods-2963108-supplementary.pdf]

**Table S1.** Analytical method validation and optimized MRM transitions of n=108 pesticides, n=18 PCBs and n=13 PAHs under analysis.

| N°. | Compound | Transition 1 (m/z) | CE 1 (eV) | Transition 2 (m/z) | CE 2 (eV) | $R^2$ | LOD (µg/Kg) | LOQ (µg/Kg) |
|---|---|---|---|---|---|---|---|---|
| **Pesticides** | | | | | | | | |
| *Carbamates* | | | | | | | | |
| **1** | (±)-Indoxacarb | 218 → 203 | 10 | 218 → 134 | 20 | 0.993 | 0.15 | 0.56 |
| **2** | Bendiocarb | 166 → 151 | 10 | 166 → 109 | 20 | 0.999 | 0.03 | 0.10 |
| **3** | Carbaryl | 144 → 115 | 20 | 115 → 89 | 20 | 0.997 | 0.09 | 0.33 |
| **4** | Carbofuran | 164 → 149 | 15 | 164 → 103 | 20 | 0.995 | 0.06 | 0.18 |
| **5** | Carbophenothion | 157 → 121 | 20 | 157 → 77 | 20 | 0.996 | 0.06 | 0.20 |
| **6** | Diethofencarb | 267 → 225 | 10 | 267 → 168 | 20 | 1.000 | 0.05 | 0.17 |
| **7** | Ethiofencarb | 168 → 107 | 10 | 168 → 77 | 25 | 0.999 | 0.08 | 0.27 |
| **8** | Furathiocarb | 194 → 105 | 20 | 194 → 165 | 15 | 0.988 | 0.06 | 0.22 |
| **9** | Phenoxycarb | 116 → 88 | 15 | 186 → 109 | 15 | 0.996 | 0.13 | 0.42 |
| **10** | Pirimicarb | 238 → 166 | 10 | 206 → 166 | 15 | 0.992 | 0.03 | 0.09 |
| *Carbamates /Acaricides* | | | | | | | | |
| **11** | Mecarbam | 296 → 196 | 10 | 296 → 168 | 10 | 0.996 | 1.16 | 4.17 |
| *Fungicides* | | | | | | | | |
| **12** | Azoxystrobin | 344 → 329 | 20 | 344 → 183 | 20 | 0.990 | 0.07 | 0.25 |
| **13** | Boscalid | 342 → 140 | 10 | 342 → 112 | 25 | 0.986 | 0.15 | 0.56 |
| **14** | Bupirimate | 208 → 165 | 15 | 108 → 140 | 15 | 0.999 | 0.96 | 3.81 |
| **15** | Captafol | 151 → 79 | 20 | 151 → 122 | 10 | 0.994 | 0.04 | 0.12 |
| **16** | Captan | 107 → 79 | 10 | 107 → 77 | 20 | 0.987 | 1.30 | 4.79 |
| **17** | Cyproconazole isomer II | 222 → 125 | 20 | 224 → 127 | 20 | 0.999 | 0.06 | 0.19 |
| **18** | Diclobutrazol | 270 → 159 | 10 | 270 → 137 | 25 | 0.962 | 0.11 | 0.30 |
| **19** | Fenarimol | 251 → 139 | 20 | 251 → 111 | 25 | 0.973 | 0.31 | 1.10 |
| **20** | Fenhexamid | 177 → 78 | 20 | 177 → 113 | 20 | 0.929 | 2.58 | 8.04 |
| **21** | Fluodioxonil | 248 → 127 | 20 | 248 → 154 | 25 | 0.965 | 0.40 | 1.27 |
| **22** | Flusilazole | 233 → 165 | 20 | 233 → 152 | 20 | 0.982 | 2.55 | 8.36 |
| **23** | Imazalil | 215 → 173 | 15 | 215 → 145 | 25 | 0.987 | 0.28 | 0.92 |
| **24** | Kresoxim methyl | 206 → 131 | 10 | 206 → 116 | 10 | 1.000 | 1.38 | 4.23 |
| **25** | Metalaxyl-M | 160 → 130 | 20 | 160 → 144 | 20 | 0.993 | 0.08 | 0.25 |
| **26** | Mepronil | 269 → 119 | 10 | 210 → 181 | 20 | 0.996 | 0.07 | 0.23 |
| **27** | Penconazole | 248 → 157 | 20 | 248 → 192 | 20 | 0.990 | 0.41 | 1.34 |
| **28** | Prochloraz | 180 → 138 | 15 | 180 → 69 | 20 | 0.990 | 1.07 | 3.05 |
| **29** | Procymidone | 283 → 96 | 10 | 285 → 96 | 15 | 0.991 | 0.13 | 0.48 |
| **30** | Pyrimethanil | 198 → 118 | 30 | 199 → 198 | 25 | 0.983 | 0.25 | 0.82 |
| **31** | Quintozen | 237 → 143 | 20 | 237 → 119 | 20 | 0.999 | 0.12 | 0.36 |
| **32** | Tebuconazole | 250 → 125 | 15 | 125 → 89 | 25 | 1.000 | 0.15 | 0.49 |
| **33** | Tolchlophos methyl | 265 → 250 | 20 | 265 → 93 | 24 | 0.966 | 0.38 | 1.26 |
| **34** | Triadimefon | 208 → 181 | 10 | 208 → 127 | 15 | 0.997 | 0.09 | 0.34 |
| **35** | Trifloxystrobin | 190 → 130 | 15 | 190 → 102 | 25 | 0.988 | 0.31 | 1.06 |
| **36** | Vinclozolin | 212 → 177 | 15 | 212 → 145 | 20 | 0.997 | 2.48 | 8.18 |
| *Herbicides* | | | | | | | | |
| **37** | Amandryn | 227 → 170 | 10 | 227 → 185 | 10 | 0.993 | 2.53 | 8.26 |
| **38** | Atrazine | 200 → 122 | 15 | 215 → 200 | 10 | 0.998 | 0.11 | 0.45 |
| **39** | Diflufenican | 266 → 183 | 25 | 246 → 218 | 25 | 0.995 | 5.09 | 14.57 |
| **40** | Linuron | 160 → 133 | 15 | 160 → 125 | 15 | 0.979 | 5.54 | 18.74 |
| **41** | Methabenzthiazuron | 164 → 136 | 15 | 127 → 109 | 20 | 0.997 | 0.06 | 0.22 |
| **42** | Oxyfluorfen | 300 → 223 | 20 | 252 → 170 | 25 | 0.981 | 0.36 | 1.38 |

| | | | | | | | | |
|---|---|---|---|---|---|---|---|---|
| **43** | Propazine | 214 → 172 | 15 | 214 → 94 | 20 | 0.973 | 0.07 | 0.20 |
| **44** | Propyzamide | 173 → 145 | 15 | 173 → 109 | 25 | 0.997 | 0.04 | 0.10 |
| **45** | Simazine | 201 → 173 | 7 | 201 → 186 | 8 | 0.999 | 0.06 | 0.22 |
| **46** | Terbuthilazine | 214 → 104 | 15 | 214 → 132 | 10 | 0.998 | 0.13 | 0.40 |
| **47** | Trifluralin | 264 → 160 | 15 | 264 → 206 | 10 | 0.975 | 5.28 | 17.89 |
| *Insect growth regulators* | | | | | | | | |
| **48** | Buprofezin | 175 → 132 | 15 | 175 → 117 | 20 | 0.952 | 0.17 | 0.63 |
| **49** | Cyromazine | 151 → 109 | 15 | 165 → 123 | 20 | 0.994 | 0.05 | 0.17 |
| **50** | Pyriproxyfen | 136 → 78 | 20 | 136 → 96 | 20 | 0.997 | 0.18 | 0.64 |
| *Organochlorine pesticides* | | | | | | | | |
| **51** | 2,4′-DDD | 235 → 165 | 20 | 237 → 165 | 20 | 0.991 | 0.09 | 0.33 |
| **52** | 2,4′-DDE | 246 → 176 | 20 | 318 → 248 | 20 | 0.963 | 0.55 | 1.62 |
| **53** | 2,4′-DDT | 235 → 165 | 20 | 237 → 165 | 20 | 0.994 | 0.26 | 0.79 |
| **54** | 4,4′-DDD | 235 → 165 | 20 | 237 → 165 | 20 | 0.999 | 0.11 | 0.35 |
| **55** | 4,4′-DDE | 246 → 176 | 30 | 318 → 248 | 30 | 0.997 | 0.16 | 0.56 |
| **56** | 4,4′-DDT | 235 → 165 | 20 | 237 → 165 | 20 | 0.999 | 2.31 | 8.17 |
| **57** | Alachlor | 188 → 160 | 15 | 161 → 146 | 15 | 0.987 | 0.04 | 0.12 |
| **58** | Aldrin | 263 → 193 | 20 | 293 → 258 | 20 | 0.992 | 0.76 | 2.88 |
| **59** | *cis*-Chlordane | 373 → 266 | 20 | 373 → 264 | 20 | 0.997 | 0.20 | 0.64 |
| **60** | Dicofol | 250 → 139 | 20 | 250 → 215 | 10 | 0.993 | 0.12 | 0.38 |
| **61** | Dieldrin | 263 → 193 | 20 | 263 → 228 | 20 | 0.992 | 0.19 | 0.73 |
| **62** | Endosulfan sulfate | 272 → 237 | 15 | 274 → 239 | 15 | 0.997 | 0.09 | 0.37 |
| **63** | Endosulfan $\alpha$ | 241 → 206 | 25 | 241 → 170 | 25 | 0.994 | 0.15 | 0.46 |
| **64** | Endosulfan β | 195 → 160 | 10 | 195 → 125 | 20 | 0.999 | 0.07 | 0.20 |
| **65** | Endrin | 263 → 193 | 20 | 281 → 245 | 15 | 0.984 | 0.13 | 0.49 |
| **66** | Methoxychlor | 227 → 169 | 20 | 227 → 141 | 25 | 0.973 | 0.14 | 0.46 |
| **67** | *trans*-Chlordane | 373 → 266 | 20 | 373 → 264 | 20 | 0.972 | 0.12 | 0.47 |
| **68** | $\alpha$-HCH | 181 → 145 | 10 | 219 → 183 | 10 | 0.988 | 0.04 | 0.13 |
| **69** | β-HCH | 181 → 145 | 15 | 219 → 183 | 10 | 0.985 | 0.22 | 0.82 |
| **70** | γ-HCH | 181 → 145 | 15 | 219 → 183 | 10 | 0.996 | 0.11 | 0.43 |
| *Organophosphorous pesticides* | | | | | | | | |
| **71** | Acephate | 136 → 94 | 10 | 136 → 119 | 8 | 0.989 | 0.33 | 1.15 |
| **72** | Andhion | 231 → 175 | 15 | 231 → 129 | 20 | 0.972 | 0.34 | 1.09 |
| **73** | Azinphos ethyl | 160 → 132 | 5 | 160 → 77 | 10 | 0.994 | 0.29 | 0.95 |
| **74** | Chlorpyriphos | 197 → 169 | 15 | 197 → 169 | 15 | 0.999 | 1.58 | 5.71 |
| **75** | Chlorpyriphos methyl | 286 → 93 | 25 | 286 → 271 | 20 | 0.984 | 0.76 | 2.78 |
| **76** | *cis*-Chlorfenvinphos | 267 → 159 | 20 | 269 → 161 | 20 | 0.984 | 0.09 | 0.31 |
| **77** | Coumaphos | 226 → 163 | 20 | 226 → 135 | 25 | 0.999 | 0.26 | 0.92 |
| **78** | Diazinon | 137 → 84 | 15 | 179 → 137 | 20 | 0.992 | 0.09 | 0.35 |
| **79** | Dimethoate | 125 → 79 | 20 | 125 → 79 | 8 | 1.000 | 0.11 | 0.37 |
| **80** | Fenamiphos | 303 → 154 | 15 | 303 → 195 | 10 | 0.988 | 0.10 | 0.33 |
| **81** | Fenchlorphos | 285 → 270 | 20 | 285 → 240 | 20 | 0.990 | 0.06 | 0.21 |
| **82** | Fenitrothion | 125 → 79 | 15 | 277 → 125 | 18 | 0.998 | 0.07 | 0.26 |
| **83** | Fenthion | 278 → 109 | 20 | 278 → 125 | 22 | 0.995 | 0.19 | 0.58 |
| **84** | Fenthion Sulfone | 310 → 105 | 20 | 310 → 109 | 30 | 0.976 | 0.74 | 2.79 |
| **85** | Fenthion Sulfoxide | 278 → 109 | 15 | 278 → 169 | 25 | 0.998 | 0.04 | 0.15 |
| **86** | Malathion | 173 → 99 | 15 | 173 → 117 | 15 | 1.000 | 0.09 | 0.28 |
| **87** | Methidathion | 145 → 85 | 10 | 145 → 58 | 20 | 0.992 | 0.07 | 0.20 |
| **88** | Omethoate | 156 → 110 | 10 | 156 → 79 | 30 | 0.994 | 0.14 | 0.45 |
| **89** | Parathion methyl | 263 → 109 | 15 | 263 → 246 | 6 | 1.000 | 0.06 | 0.15 |
| **90** | Phenthoate | 274 → 125 | 15 | 274 → 121 | 15 | 0.999 | 0.11 | 0.33 |

| | | | | | | | | |
|---|---|---|---|---|---|---|---|---|
| **91** | Phosalone | 182 → 111 | 20 | 182 → 75 | 30 | 0.995 | 0.06 | 0.17 |
| **92** | Phosmet | 160 → 77 | 25 | 160 → 133 | 15 | 0.997 | 0.04 | 0.15 |
| **93** | Phoxim | 109 → 81 | 15 | 109 → 91 | 15 | 1.000 | 0.10 | 0.34 |
| **94** | Quinalphos | 146 → 118 | 15 | 146 → 91 | 30 | 0.994 | 0.07 | 0.22 |
| **95** | *trans*-Chlorfenvinphos | 267 → 159 | 20 | 269 → 161 | 20 | 0.996 | 0.08 | 0.29 |
| **96** | Triphenyl phosphate | 325 → 169 | 20 | 325 → 77 | 25 | 0.998 | 0.09 | 0.34 |
| *Pyrethroid insecticides* | | | | | | | | |
| **97** | Carbophenothion | 157 → 121 | 20 | 157 → 77 | 20 | 0.999 | 0.10 | 0.37 |
| **98** | Pirimiphos-methyl | 290 → 125 | 15 | 290 → 151 | 15 | 0.992 | 0.14 | 0.46 |
| **99** | *cis*-Fluvalinate | 250 → 55 | 15 | 252 → 55 | 20 | 0.997 | 0.39 | 1.18 |
| **100** | *cis*-Permethrin | 183 → 153 | 15 | 183 → 168 | 15 | 1.000 | 0.06 | 0.20 |
| **101** | Cypermethrin isomer I | 181 → 152 | 20 | 163 → 91 | 15 | 0.995 | 0.06 | 0.22 |
| **102** | Cypermethrin isomer II | 181 → 152 | 20 | 163 → 91 | 15 | 0.998 | 0.09 | 0.30 |
| **103** | Cypermethrin isomer III | 181 → 152 | 20 | 163 → 91 | 15 | 0.997 | 0.07 | 0.24 |
| **104** | Deltamethrin | 181 → 152 | 20 | 253 → 93 | 15 | 1.000 | 0.08 | 0.27 |
| **105** | *trans*-Fluvalinate | 250 → 55 | 15 | 252 → 55 | 20 | 0.996 | 0.08 | 0.29 |
| **106** | *trans*-Permethrin | 183 → 153 | 20 | 183 → 168 | 20 | 0.984 | 0.10 | 0.36 |
| **107** | Λ-Cyhalothrin | 181 → 152 | 25 | 197 → 141 | 10 | 0.989 | 0.13 | 0.41 |
| *Synergists* | | | | | | | | |
| **108** | Piperonyl butoxide | 176 → 131 | 15 | 176 → 103 | 20 | 0.999 | 0.05 | 0.18 |
| ***PCBs*** | | | | | | | | |
| **1** | PCB28 | 256 → 186 | 15 | 258 → 186 | 15 | 1.000 | 0.15 | 0.48 |
| **2** | PCB52 | 290 → 220 | 15 | 292 → 222 | 15 | 0.998 | 0.07 | 0.22 |
| **3** | PCB77 | 290 → 220 | 20 | 292 → 222 | 20 | 1.000 | 0.12 | 0.41 |
| **4** | PCB81 | 290 → 220 | 20 | 292 → 222 | 20 | 0.999 | 0.06 | 0.22 |
| **5** | PCB101 | 324 → 254 | 20 | 326 → 256 | 20 | 0.997 | 0.08 | 0.24 |
| **6** | PCB105 | 324 → 254 | 20 | 326 → 256 | 20 | 0.990 | 0.04 | 0.15 |
| **7** | PCB114 | 324 → 254 | 20 | 326 → 256 | 20 | 0.992 | 0.12 | 0.39 |
| **8** | PCB118 | 324 → 254 | 20 | 326 → 256 | 20 | 0.998 | 0.12 | 0.35 |
| **9** | PCB123 | 324 → 254 | 20 | 326 → 256 | 20 | 1.000 | 0.04 | 0.14 |
| **10** | PCB126 | 324 → 254 | 20 | 326 → 256 | 20 | 0.987 | 0.03 | 0.10 |
| **11** | PCB138 | 360 → 290 | 25 | 362 → 292 | 25 | 0.980 | 0.10 | 0.36 |
| **12** | PCB153 | 360 → 290 | 25 | 362 → 292 | 25 | 0.999 | 0.05 | 0.19 |
| **13** | PCB156 | 360 → 290 | 30 | 362 → 292 | 30 | 0.998 | 0.04 | 0.15 |
| **14** | PCB157 | 360 → 290 | 30 | 362 → 292 | 30 | 0.975 | 0.16 | 0.55 |
| **15** | PCB167 | 360 → 290 | 30 | 362 → 292 | 30 | 0.997 | 0.11 | 0.43 |
| **16** | PCB169 | 360 → 290 | 30 | 362 → 292 | 30 | 0.995 | 0.07 | 0.24 |
| **17** | PCB180 | 394 → 324 | 20 | 396 → 326 | 20 | 0.993 | 0.03 | 0.11 |
| **18** | PCB189 | 394 → 324 | 25 | 396 → 326 | 25 | 0.999 | 0.04 | 0.12 |
| ***PAHs*** | | | | | | | | |
| **1** | Acenaphthylene | 152 → 126 | 30 | 152 → 102 | 30 | 0.992 | 0.06 | 0.19 |
| **2** | Anthracene | 178 → 152 | 25 | 176 → 150 | 25 | 0.993 | 0.07 | 0.20 |
| **3** | Benzo[a]anthracene | 228 → 226 | 30 | 228 → 202 | 20 | 0.991 | 0.06 | 0.21 |
| **4** | Benzo[a]pyrene | 252 → 250 | 35 | 252 → 226 | 20 | 0.998 | 0.08 | 0.25 |
| **5** | Benzo[b]fluoranthene | 252 → 250 | 35 | 126 → 113 | 10 | 0.989 | 0.28 | 0.89 |
| **6** | Benzo[g,h,i]perylene | 276 → 274 | 45 | 276 → 272 | 50 | 0.999 | 0.07 | 0.21 |
| **7** | Benzo[k]fluoranthene | 252 → 250 | 35 | 126 → 113 | 10 | 0.994 | 0.14 | 0.48 |
| **8** | Chrysene | 228 → 226 | 30 | 228 → 202 | 20 | 0.990 | 0.06 | 0.17 |
| **9** | Dibenzo[a.h]anthracene | 278 → 276 | 30 | 278 → 252 | 20 | 0.998 | 0.06 | 0.23 |
| **10** | Fluorene | 166 → 165 | 15 | 165 → 164 | 20 | 0.996 | 0.04 | 0.15 |
| **11** | Indeno[1,2,3-cd]pyrene | 276 → 274 | 30 | 137 → 136 | 15 | 0.995 | 0.09 | 0.29 |

| | | | | | | | | |
|---|---|---|---|---|---|---|---|---|
| **12** | Phenanthrene | 178 → 152 | 25 | 176 → 150 | 25 | 1.000 | 0.05 | 0.16 |
| **13** | Pyrene | 202 → 200 | 20 | 202 → 152 | 30 | 0.996 | 1.28 | 4.19 |

CE, Collision Energy; $R^2$, determination coefficient; LOD, Limit of Detection; LOQ, Limit of Quantification

**Table S2.** Analytical method validation and monitored ions of n=10 PAEs and n=8 NPPs under analysis.

| *N°.* | *Compound* | *Abbreviation* | *Monitored ions (m/z)* | $R^2$ | *LOD (mg/Kg)* | *LOQ (mg/Kg)* |
|---|---|---|---|---|---|---|
| | | | ***PAEs*** | | | |
| **1** | Dimethyl Phthalate | DMP | 163, 92, 164 | 0.996 | 0.007 | 0.021 |
| **2** | Diethyl Phthalate | DEP | 149, 177, 176 | 0.995 | 0.003 | 0.010 |
| **3** | Dipropyl Phthalate | DPrP | 149, 150, 209 | 0.991 | 0.004 | 0.013 |
| **4** | Dibutyl Phthalate | DBP | 149, 150, 223 | 0.990 | 0.006 | 0.021 |
| **5** | Diisobutyl Phthalate | DiBP | 149, 150, 223 | 0.997 | 0.007 | 0.027 |
| **6** | Butyl Benzyl Phthalate | BBP | 149, 91, 206 | 0.999 | 0.004 | 0.012 |
| **7** | Diphenyl Phthalate | DPhP | 225, 226, 104 | 0.992 | 0.018 | 0.062 |
| **8** | Dicyclohexyl Phthalate | DcHexP | 149, 167, 150 | 0.999 | 0.028 | 0.087 |
| **9** | Diheptyl Phthalate | DHepP | 149, 99, 265 | 0.997 | 0.177 | 0.555 |
| **10** | Di(2-ethylhexyl) Phthalate | DEHP | 149, 167, 279 | 0.999 | 0.007 | 0.025 |
| | | | ***NPPs*** | | | |
| **1** | Dimethyl Adipate | DMA | 114, 101, 111 | 0.998 | 0.011 | 0.033 |
| **2** | Diethyl Adipate | DEA | 111, 157, 128 | 0.992 | 0.003 | 0.010 |
| **3** | Benzyl Benzoate | BB | 105, 91, 212 | 0.987 | 0.012 | 0.043 |
| **4** | Dibutyl Adipate | DBA | 129, 185, 111 | 0.989 | 0.023 | 0.071 |
| **5** | Diisobutyl Adipate | DiBA | 129, 185, 111 | 0.993 | 0.009 | 0.029 |
| **6** | Di(2-ethylhexyl) Adipate | DEHA | 129, 112, 147 | 0.982 | 0.014 | 0.044 |
| **7** | Di(2-ethylhexyl) Terephthalate | DEHT | 149, 112, 261 | 0.988 | 0.009 | 0.033 |
| **8** | Di(2-ethylhexyl) Sebacate | DEHS | 185, 149, 112 | 0.997 | 0.048 | 0.182 |

$R^2$, coefficient of determination; LOD, Limit of Detection; LOQ, Limit of Quantification.
Underlined ions were considered for quantitative analysis.

**Table S3.** Analytical method validation and MS/MS condition of n=9 BPs under analysis.

| *N°.* | *Compound* | *Abbreviation* | *Transition 1 (m/z)* | *CE 1 (eV)* | *Transition 2 (m/z)* | *CE 2 (eV)* | $R^2$ | *LOD (µg/Kg)* | *LOQ (µg/Kg)* |
|---|---|---|---|---|---|---|---|---|---|
| **1** | 4,4′-Sulfonyldiphenol | BPS | 249.2 → 107.9 | 15 | 249.27 → 156.0 | 12 | 0.998 | 0.297 | 1.142 |
| **2** | 4,4′-Methylenediphenol | BPF | 199.2 → 93.1 | 13 | 199.23 → 105.1 | 14 | 0.993 | 0.385 | 1.362 |
| **3** | 1,1-Bis(4-hydroxyphenyl) ethane | BPE | 213.3 → 198.0 | 38 | 213.26 →194.9 | 40 | 0.992 | 0.328 | 1.168 |
| **4** | 4,4'-(propan-2.2-diyl) diphenol | BPA | 227.3 → 212.1 | 17 | 227.29 → 133.0 | 18 | 0.988 | 0.431 | 1.457 |
| **5** | 4-[2-(4-hydroxyphenyl) butan-2-yl] phenol | BPB | 241.3 → 212.0 | 20 | 241.31 → 211.0 | 21 | 0.995 | 0.289 | 0.945 |
| **6** | 2,2-Bis(4-hydroxyphenyl) hexafluoropropane | BPAF | 335.3 → 265.0 | 35 | 335.30 → 177.0 | 33 | 0.996 | 0.276 | 0.839 |
| **7** | 1,1-Bis(4-hydroxyphenyl)-1-phenyl-ethane | BPAP | 289.4 → 274.1 | 10 | 289.36 → 273.1 | 10 | 0.994 | 0.462 | 1.591 |
| **8** | 1,1-Bis(4-hydroxyphenyl)-cyclohexane | BPZ | 267.3 → 145.0 | 17 | 267.30 → 173.1 | 18 | 0.997 | 0.435 | 1.483 |
| **9** | 1,4-Bis(2-(4-hydroxyphenyl)-2-propyl)benzene | BPP | 345.5 → 330.1 | 33 | 345.46 → 133.1 | 34 | 0.989 | 0.414 | 1.436 |

CE, Collision Energy; $R^2$, coefficient of determination; LOD, Limit of Detection; LOQ, Limit of Quantification.
